# How climate change and wildlife management affect population structure in wild boars

**DOI:** 10.1038/s41598-020-64216-9

**Published:** 2020-04-29

**Authors:** Sebastian G. Vetter, Zsófia Puskas, Claudia Bieber, Thomas Ruf

**Affiliations:** University of Veterinary Medicine, Vienna, Department of Interdisciplinary Life Sciences, Research Institute of Wildlife Ecology, Savoyenstr. 1, 1160 Vienna, Austria

**Keywords:** Evolutionary ecology, Population dynamics

## Abstract

Global climate change affects many species and contributes to the exceptional population growth of wild boar populations and thus to increasing human-wildlife conflicts. To investigate the impact of climate change on wild boar populations we extended existing models on population dynamics. We included for the first time different juvenile conditions to account for long-lasting effects of juvenile body mass on adult body mass and reproductive success. Our analysis shows that incorporating phenotypes, like body mass differences within age classes, has strong effects on projected population growth rates, population structures and the relative importance of certain vital rates. Our models indicated that an increase in winter temperatures and food availability will cause a decrease in mean body mass and litter size within Central European wild boar populations. We further analysed different hunting regimes to identify their effects on the population structure as well as their efficiency in limiting population growth. While targeting juveniles had the lowest effect on population structure, such strategies are, however, rather ineffective. In contrast, culling predominantly yearlings seems very effective. Despite being equally effective, only focusing on adults will not result in a reduction of population size due to their low proportion within populations.

## Introduction

Climate change affects population growth as well as demography and population structure in various species e.g.^1–4^. While many species decline in abundance and distribution others cope well with climate change and thrive^1,5,6^. The latter typically reach high population densities, expand their distribution range and thus might cause severe human-wildlife conflictssuch as agricultural damage, the spread of diseases, negative effects on other species, or an increased risk of traffic collisions^7^.

The wild boar *Sus scrofa*is among the clear winners of global climate change. The tremendous growth of wild boar populations over the past decades could already be linked to improvements in food availability and winter weather conditions caused by climate change^8–13^. Apart from a few regions, negative effects like density dependence or increasingly hotter summers are unlikely to noticeably limit this growth in the near future^13^. With ongoing climate change, the current population growth is therefore likely to continue or even to accelerate.

Wild boars have a tremendous reproductive potential with one sow being able to give birth to up to 14 offspring in a single litter^14–17^. At the same time reproduction is also highly variable in wild boar with respect to litter size and also offspring body mass can vary strongly between years, within years, and even within litters^14,17–19^. Juvenile body mass, however, affects adult body mass and reproductive success in wild boar, as in other ungulates^20,21^. In previous models of wild boar population dynamics these long-lasting effects of juvenile body mass have not been considered^14,16,22^.

Especially smaller juveniles, which have high rates of heat loss^23,24^, will benefit from improving environmental conditions, i.e. increasing winter temperatures and food availability, and contribute disproportionally to population growth^13^. Thus, climate change most likely also affects population structure and phenotypes in wild boar, like in other species e.g.^1,6,25^. In order to investigate how favourable conditions affect wild boar population structure and dynamics we extended and refined previous population models^14,16,22^ and developed an age-body mass hybrid Leslie matrix model^26^.

Expanding our knowledge on population responses to changing environmental conditions is not only important for our understanding of how different species react to climate change but is also essential for effective management strategies. In wild boars, improved population control strategies are urgently required. First, to curtail strong population growth, which is increasing human-wildlife conflicts and ecological threats (e.g.^27–29^). Second, because guidelines recommend that if African swine fever is diagnosed nearby a population, a reduction of the wild boar population by 80% is requiredto stop the spread of the disease^30^. Management of this species via traditional hunting practices in Europe, however, seems increasingly difficult^9^.

Under good environmental conditions, juvenile survival has a particularly strong impact on wild boar population growth^16^. This led to the suggestion that harvesting female juveniles will have the strongest effect on population growth rates^16^. However, due to the large absolute number of juveniles and the indistinguishability of sexes from a distance at that age, such strategies are known to be extremely time-consuming. To identify the most effective hunting strategies we modelled a variety of different culling regimes. Further, we calculated the relative effect of the harvest of a single individual of each class, with respect to population growth, to investigate how efficient different regimes of selective hunting are with respect to population control.

Although some studies investigated how selective harvest affects wild boar population growth^14,16,22^ data on how population structure is altered are still lacking for this species. Findings on the effects of culling regimes from other species like deer or bovids^31–33^, however, cannot be easily applied to wild boars because of their very special population dynamics compared to similar sized ungulates^14^. We therefore additionally modelled how different culling regimes may affect population structure under environmental conditions favouring population growth.

Previous findings show that younger, smaller individuals gain importance under increasing winter temperatures and food availability^16^. We therefore hypothesised that, even within age classes, smaller individuals will become increasingly important regarding population growth and constitute a higher proportion within the population in response to improving environmental conditions. Given the naturally high proportions of juveniles within wild boar populations^34^, we further hypothesised that culling regimes focusing on this age class will disrupt the natural population structure the least. However, as reproductive output increases with age, e.g.^34,35^, we expected culling regimes focusing more on older age classes to be more efficient in limiting wild boar population growth.

## Material and Methods

### Life cycle and matrix model

**Figure 1:**
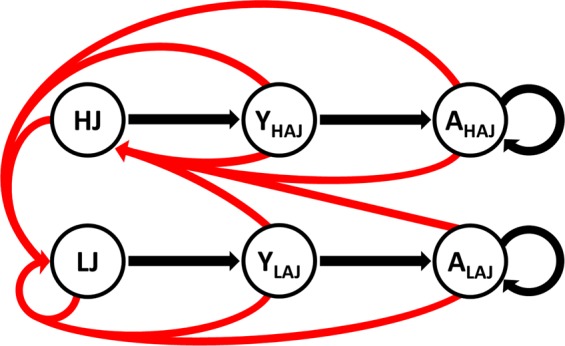
Life cycle model with three age classes of female wild boars (i.e., juveniles, yearlings and adults). These were additionally divided in two groups respectively in order to incorporate the long lasting effects of juvenile body mass. Black arrows indicate survival, red arrows indicate fecundity.

In order to model effects of environmental changes and selective hunting on wild boar population structure, we used a hybrid Leslie matrix population model (Fig. 1). This model combined an age-class model with a two-level phenotype variable based on juvenile body mass resulting in six female classes (i.e., heavy and light juveniles, yearlings that have been heavy or light and adults that have been heavy or light as juveniles). For an overview on the abbreviations used for different classes see Table 1. In accordance with previous publications, juveniles were categorised into light or heavy based on a threshold body mass of 30 kg live weight^35,36^ (for detailed information see Supplementary Information 1). A one-year autumn-to-autumn projection interval was used. In wild boars, juveniles can be born until late summer^37,38^. Consequently, this interval included the extended parturition season and yielded a complete post-reproduction model. This results in a more accurate number of offspring present in autumn, which is most important for managers as the main hunting seasons for wild boar in Central Europe are autumn and winter. Further, juvenile body mass in autumn is already informative regarding the reproductive potential within the upcoming reproductive season^35,36^.

**Table 1:** Abbreviations for classes of female wild boar.

Abbreviation	Class
LJ	light juveniles
HJ	heavy juveniles
Y_LAJ_	yearlings that were light juveniles
Y_HAJ_	yearlings that were heavy juveniles
A_LAJ_	adults that were light juveniles
A_HAJ_	adults that were heavy juveniles
LAJ	Y_LAJ_ + A_LAJ_
HAJ	Y_HAJ_ + A_HAJ_

Because of this projection interval, age class transition as well as parturition occurred in the middle of the interval for most of the individuals. Consequently, the computed fecundity incorporated winter survival of the reproducing female as well as summer survival of the produced female offspring. Given a balanced sex ratio in wild boar^34^ this resulted in the following equation for fecundity:

*F* = winter survival * breeding probability * average number of offspring * offspring summer survival * 0.5

### Modelling environmental conditions

To model effects of changing environmental conditions on population structure and phenotype, the model was parametrised for two scenarios: favourable (tree mast and mild winter) and unfavourable (no mast and severe winter) environmental conditions. Survival and fecundity rates for parametrisation of the two models were taken from the literature (Table 2; for detailed information see Supplementary Information 1). Weight class specific fecundities and relative contributions to HJ (heavy juveniles) and LJ (light juveniles, all abbreviations are given in Table 1) were calculated using our own data on reproduction of yearlings heavy or light as juveniles (n[Y_LAJ_]= 20, n[Y_HAJ_]= 21, n[Offspring]= 112) and adult sows heavy or light as juveniles (n[A_LAJ_]= 23, n[A_HAJ_]= 20, n[Offspring]= 112) kept under semi-natural conditions (for detailed information see Supplementary Information 1). No data were available for seasonal survival of yearling and adult wild boar and for the differences in winter survival between HJ and LJ. The main natural cause of death among older wild boar classes is starvation^39^, which occurs mostly in winter when food availability is lowest. Consequently, a high summer survival of 95% was assumed for yearlings and adults. Further, smaller individuals are more susceptible to cold temperatures because of a lower body surface to volume ratio and the resulting higher energy demands for thermoregulation^23,24^. It therefore seems plausible that LJ have lower survival rates than HJ. Thus, winter survival was assumed to be 10% higher and lower for HJ and LJ, respectively, compared to the averages for all juveniles under favourable and unfavourable conditions. The effects of these assumptions on the model outcome were tested by altering the respective percentages (Supplementary Information S2).

**Table 2:** Vital rates used for each class and environmental condition.

Vital rate	Conditions	LJ	HJ	Y_LAJ_	Y_HAJ_	A_LAJ_	A_HAJ_	References
Yearly survival (S_y_)	favourable	0.42^a^	0.6^a^	0.60^b^	0.60^b^	0.71^b^	0.71^b^	^a^assumed*^b^Briedermann^34^
	unfavourable	0.15^a^	0.35^a^	0.31^b^	0.31^b^	0.58^b^	0.58^b^	
Summer survival (S_s_)		0.73^c^	0.73^c^	0.95^a^	0.95^a^	0.95^a^	0.95^a^	^a^assumed* ^c^Fruzinski^56^
Winter survival (S_w_)	favourable	0.58	0.85	0.63	0.63	0.75	0.75	S_w_ = S_y_/S_s_
	unfavourable	0.21	0.48	0.33	0.33	0.61	0.61	
Mean No. of foetuses	favourable	3.88	4.90	5.65	7.13	6.36	7.41	Briedermann^34^; own data
	unfavourable	0	3.20	4.55	5.75	5.80	6.76	
Breeding probability	favourable	0.10^d^	0.60^e^	1.00^e^	1.00^e^	1.00^e^	1.00^e^	^e^Gethöffer, *et al*.^35^ ^d^Servanty *et al*.^17^
	unfavourable	0^d^	0.43^e^	1.00^e^	1.00^e^	1.00^e^	1.00^e^	
Intra uterine mortality		0.11		0.18		0.06		Gethöffer, *et al*.^35^
Fecundity (F)	favourable	0.073	0.812	1.065	1.344	1.637	1.907	Calculated
	unfavourable	0.000	0.215	0.449	0.568	1.214	1.415	

^*^The effect of the assumptions on the model outcome and the conclusions was tested in separate analyses. Neither assumption affected the conclusion we drew from the model outcomes (see text and Supplementary Information 2 for more information).

Intermediate environmental conditions (e.g., mast and severe winter) were not modelled due to poor data availability and the fact that sufficient food availability in winter compensates for negative effects of severe winters^13^. Only data from Central European populations were considered for model parametrisation as Mediterranean populations are considerably different with respect to body mass, litter size and other life history parameters^13,40^.

The resulting projection matrices were analysed with respect to stable stage structure, sensitivities and elasticities^26^, i.e., the absolute or proportional effect of each matrix element on asymptotic population growth rate (λ), respectively^41^.

All statistical analyses were computed in R Version 3.3.2;^42^ using the R-package ‘popbio’ Version 2.43;^41^.

### Modellingculling regimes

In Central Europe, wild boar hunting usually concentrates in the season of late autumn until the end of January^35,36^. Therefore, summer hunting mortality was ignored in the present model with hunting mortality only adding to winter but not to summer mortality. Differences in juvenile body mass (heavy or light juveniles) remain visible in autumn and winter, i.e. seasons very close to the time of the classification of juveniles into the two weight classes. Therefore, selective harvest within juveniles was considered in the modelled hunting scenarios. In contrast, a reliable classification of yearlings and adults based on their juvenile body mass is less likely. Despite slight long-term effects of juvenile on adult body mass, additional factors might have affected growth or body condition in these classes. Therefore, hunting mortalities of HAJ or LAJ females (abbreviations see Table 1) were considered to be proportional to their respective numbers within the age classes. This resulted in four different hunting classes: light juveniles, heavy juveniles, yearlings, and adults.

Series of hunting mortality rates (HMR) for each class were created ranging from 0 (none harvested) to 1 (all harvested) at 0.05 intervals. To investigate the effect on population growth and structure we focused on the following culling regimes: *(i)* a totally unselective hunt (HMR of all age classes are the same);* (ii) *a highly selective hunt on only one of the four classes with HMR = 0 for all other classes, to identify the effect of removing individuals of each age class separately, and *(iii) *three culling regimes mimicking natural predation with a low HMR of 25% for classes older than one year (as recommended in^34,43^)and differential selection among juveniles (i.e., HMR of juveniles ranging from 0 to 1 with NH1: no selection among juveniles, NH2: selective removal of heavy juveniles only, and NH3: selective removal of light juveniles only).

**Figure 2:**
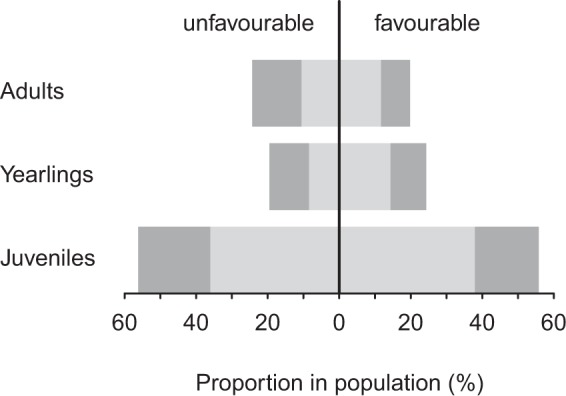
Stable stage distribution under favourable and unfavourable environmental conditions without hunting. We refer here to “stage” since we considered age and body mass together in our modelling procedure. Thus light juveniles as well as yearlings and adults, which have been light as juveniles are displayed in light grey, heavy juveniles as well as yearlings and adults, which have been heavy as juveniles are displayed in dark grey. Please note that there can be more adults than yearlings because adults remain several years within this age class whereas yearlings and juveniles remain only one year in the respective age class.

To account for improving environmental conditions^13^ the model parametrised for favourable conditions was used to model the effects of the different culling regimes. This was done by multiplying both fecundities and transition probabilities of this parametrisation with hunting survival (1–HMR). To compare the efficiency of different strategies, hunting mortality scenarios which resulted in a stable population (λ ≈ 1) were translated into absolute numbers of females to be harvested. For this we used an idealised population of 501 females according to the stable stage structure found without hunting under favourable conditions (Fig. 2).

To ease decision making for hunters, the relative impact of a single harvested individual with respect to population growth was calculated. For this, survival sensitivities were weighed according to the proportion of the respective class in the stable stage distribution and corrected for discriminability of two factors: First, differences regarding the juvenile body mass in adults and yearlings are not easily detectable. Therefore, the means of the weighted sensitivities of the two groups (i.e., HAJ and LAJ) were used for all yearling and adult females, respectively. Second, sexual differences in juveniles are not easily detectable from a distance. Thus, given a balanced sex ratio in wild boar juveniles^34^, weighted sensitivities of both juvenile classes (i.e. HJ and LJ) were multiplied by 0.5. All values were then standardised using the lowest resulting value of corrected weighed sensitivity (Supplementary Table S2).

## Results

Importantly, our models were very robust towards changes to both assumptions, i.e., summer survival of yearlings and adults and differences in survival between HJ and LJ (abbreviations see Table 1). No qualitative changes to the model outcome could be identified except for two scenarios: First, under the unlikely assumption that body mass would not affect juvenile survival under unfavourable environmental conditions (Fig. S8, S10; Supplementary Information 2); Second, if the difference in survival between LJ and HJ fell below 20%, the proportions of LAJ and HAJ females within the population could be affected under unfavourable conditions (Fig. S12; Supplementary Information 2). However, none of these scenarios affected the conclusions from the models that we outline below. For more detailed information on the results of the model validation see Supplementary Information 2.

### Effects of changing environmental conditions

The models revealed an asymptotic growth rate of λ = 1.44 under favourable conditions and of λ = 0.83 under unfavourable conditions. In both conditions, juveniles in general and especially light juveniles provided the highest proportion of females in autumn (Fig. 2). Under favourable conditions the proportion of LJ within this age class increased slightly compared with unfavourable conditions (Fig. 2). This trend became more obvious for yearlings and adults with LAJ females dominating these age classes under favourable conditions. Under unfavourable conditions HAJ females dominated their age classes. There were more yearlings than adults under favourable, but vice versa under unfavourable conditions (Fig. 2).

Interestingly, there was a marked crossover in the ranking of elasticities, i.e. the relative contribution of different vital rates to population growth. Juvenile survival (of both, LJ and HJ) was the main driver of population growth under favourable environmental conditions. The maximum elasticity changed, however, from juvenile to adult survival under poor conditions (Table 3). This predominant role of adult survival in unfavourable conditions was almost solely caused by survival of HAJ females, with an elasticity of 0.32, that was 4 times higher than that of LAJ females (0.08, Table 3).

**Table 3:** Sensitivities and elasticities of vital rates under favourable (i.e., high food availability and winter temperatures) and unfavourable (i.e., low food availability and winter temperatures) environmental conditions.

	Sensitivity		Elasticity	
	unfavourable	favourable	unfavourable	favourable
S(LJ)	0.2049	0.3931	0.0480	**0.1487**
S(HJ)	0.2864	0.2726	**0.1571**	**0.1524**
S(Y_LAJ_)	0.0923	0.1921	0.0348	0.0796
S(Y_HAJ_)	0.3744	0.2357	**0.1412**	0.0977
S(A_LAJ_)	0.1153	0.1565	0.0804	0.0769
S(A_HAJ_)	0.4674	0.1921	**0.3262**	0.0944
F(LJ)	0.0000	0.1565	0.0000	0.0079
F(HJ)	0.0268	0.0737	0.0069	0.0414
F(Y_LAJ_-LJ)	0.0113	0.0592	0.0047	0.0281
F(Y_LAJ_-HJ)	0.0689	0.1557	0.0085	0.0410
F(Y_HAJ_-LJ)	0.0147	0.0412	0.0089	0.0283
F(Y_HAJ_-HJ)	0.0900	0.1083	0.0070	0.0264
F(A_LAJ_-LJ)	0.0141	0.0482	0.0177	0.0393
F(A_LAJ_-HJ)	0.0861	0.1269	0.0171	0.0403
F(A_HAJ_-LJ)	0.0184	0.0336	0.0098	0.0116
F(A_HAJ_-HJ)	0.1124	0.0883	**0.1315**	0.0861

Vital rates are survival (S) and fecundity (F) of all wild boar classes included in the model. Yearlings and adults can contribute to both heavy and light juveniles; the contribution is signed by a hyphen (e.g., Y_LAJ_-HJ). Elasticity values over 0.1 are highlighted in bold to indicate the largest effects.

The sum of elasticities of survival and fecundity of LJ and LAJ females amounted to only 0.21 in poor conditions while this value increased to 0.46 under favourable conditions (Table 3). Under unfavourable conditions, elasticities of survival and fecundity of A_HAJ_ summed up to 0.46 (Table 3). Fecundities showed generally lower elasticities compared to survival, with the production of heavy offspring by A_HAJ_ showing the highest elasticity under both conditions (0.13 and 0.09 respectively, Table 3).

### Effects of culling regimes

**Figure 3:**
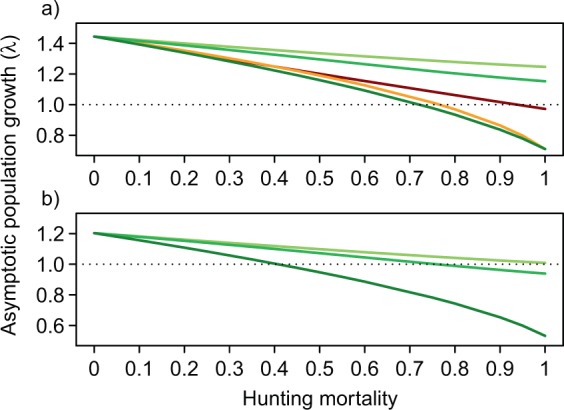
Asymptotic population growth rates (λ) of modelled wild boar populations in dependence of different hunting mortalities. (**a**) Shows the effect of pure strategies with only light juveniles (light green), heavy juveniles (medium green), juveniles non-selectively (darkgreen), yearlings (orange) and adults (red) being removed, respectively in varying proportions. (**b**) Shows the effect of the natural hunting models, i.e., the effect of hunting mortality of light juveniles (light green), heavy juveniles (medium green), or of non-selective removal of juveniles (dark green) on population growth rate given a 25% hunting mortality for yearlings and adults. In both plots the dotted line at λ = 1 indicates a stable population.

Assuming complete unselective hunting, at least 30% of the population needed to be harvested in each age class under favourable environmental conditions to prevent the population from growing (i.e., λ ≈ 1). Harvesting adult sows could stop population growth (i.e., λ = 0.97) but only if 100% of this class was harvested (Fig. 3a; Table 4). Harvesting only yearling sows yielded a stable population when about 80% of the yearlings were harvested (Fig. 3a). Harvesting either all light or all heavy juveniles exclusively did not result in a reduction of the population and λ never decreased below 1. Harvesting 75% of all juveniles irrespective of their body mass resulted in a stable population (Fig. 3a; Table 4).

**Table 4:** Modelled management scenarios that yielding a population growth rate of λ ≈ 1 (i.e., a stable population).

Management scenario		LJ	HJ	Yearlings	Adults	Sum	λ
		*(190)*	*(89)*	*(122)*	*(100)*	*(501)*	
unselective	HMR (%)	30	30	30	30		1.01
	No.	57	27	37	30	151	
NH1	HMR (%)	40	40	25	25		1.00
	No.	76	36	41	25	168	
NH2	HMR (%)	0	80	25	25		0.99
	No.	0	71	41	25	137	
NH3	HMR (%)	100	0	25	25		1.00
	No.	190	0	41	25	246	
Juveniles (unselectively)	HMR (%)	75	75	0	0		0.98
	No.	142	67	0	0	209	
Light juveniles	HMR (%)	100	0	0	0		1.25
	No.	190	0	0	0	190	
Heavy juveniles	HMR (%)	0	100	0	0		1.15
	No.	0	89	0	0	89	
Yearlings	HMR (%)	0	0	80	0		0.97
	No.	0	0	98	0	98	
Adults	HMR (%)	0	0	0	100		0.97
	No.	0	0	0	100	100	

Harvesting classes are light (LJ) and heavy juveniles (HJ), yearlings and adults. The number of animals within an exemplary population structured according to the stable stage distribution identified under favourable conditions without hunting mortality is given in parenthesis. For each management-scenario the proportion (i.e., the hunting mortality rate, HMR) and the actual numbers of individuals that need to be harvested (No.) to achieve a stable population (i.e., λ ≈ 1) are given as well as the overall sum of individuals over all classes and the exact λ under the respective scenario. The management scenarios light juveniles and heavy juveniles did not result in a stable population even when 100% of the respective individuals were harvested (i.e., λ ≫ 1).

By harvesting adults and yearlings at a low rate of 25% and selectively harvesting light juveniles no population reduction could be achieved (Table 4, NH3). Applying this natural hunting model, mimicking natural predation^36,45^, in combination with the selective harvest of heavy juveniles caused a population decline when 80% of heavy juveniles were removed (Fig. 3b; Table 4, NH2). Harvesting juveniles unselectively under such a scenario would yield a stable population when 40% of all female juveniles were removed (Fig. 3b; Table 4, NH1).

Translating these findings into actual numbers, based on a hypothetical population of 501 females, revealed that, with an entirely non-selective harvest, 151 females need to be removed to keep the population stable (Table 4). The higher the hunting pressure on older age classes (i.e., yearlings and adults) the lower the total harvest required to prevent the population from growing (Table 4).

**Figure 4:**
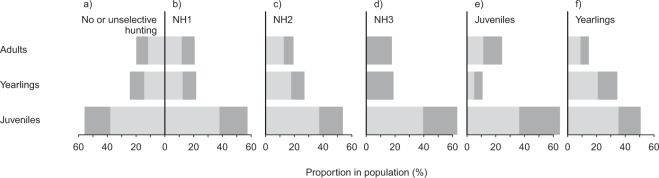
Stable stage distribution of different management scenarios: (**a**) without hunting or under an entirely unselective management scenario; (**b–d**) according to the natural hunting model with 25% hunting mortality for adults and yearlings and (**b**) non-selective removal of 40% of juveniles (NH1), (**c**) selective removal of 80% of heavy (NH2) or (**d**) 100% of light juveniles (NH3); (**e**) harvesting only juveniles with 75% of juveniles being removed non-selectively; and (**f**) harvesting only yearlings with 80% of the yearlings being removed non-selectively. Selective harvest of heavy juveniles, light juveniles and adults only cannot result in an effective population reduction (Table 3). These modelled management scenarios are therefore not shown here. Light juveniles as well as LAJ females are indicated in light grey, heavy juveniles and HAJ females in dark grey.

Compared to an entirely unselective harvest (i.e., culling 30% in each age class, Fig. 4a), the natural hunting models (NH1-3, Fig. 4b–d) showed slight increases in the proportion of adult sows within populations but overall they affected the naturally occurring stage distribution the least. Culling regimes with a high proportion of harvested light juveniles (NH3 and Juveniles) increased the proportion of juveniles to more than 60% in the stable stage distribution. This was mainly due to an increased proportion of individuals that were heavy juveniles (Fig. 4d,e). A high hunting pressure on juveniles only caused a strong decrease in the proportion of yearling females and an increase in the proportion of adult females (Fig. 4e). Harvesting exclusively yearlings, in contrast, led to a decreased proportion of juveniles and adults in the stable stage distribution (Fig. 4f).

Harvesting a single heavy juvenile affected λ 1.5 times stronger compared with culling a single light juvenile. A similar difference also exists in older age classes where HAJ had a 1.8 times higher weighted sensitivity compared with LAJ, in both age classes, yearlings and adults. Averaging the values of HAJ and LAJ females per age class revealed that in both age classes, i.e. yearlings and adults, the effect of removing one female on λ was 3.6 times higher than the effect of removing a light juvenile. For details on the calculation of the efficiency of harvesting each age class see Supplementary Table S2.

## Discussion

### Effect of improving environmental conditions

The present model shows that under unfavourable conditions HAJ females dominate within their age class if the difference in survival between heavy and light juveniles was 20% or more. Although there is no empirical data on body mass dependent winter survival in wild boars, studies on other species show that lighter individuals typically have lower survival rates e.g.^44–46^. This might be caused by energy constraints of leaner individuals or by effects of a limited thermoregulatory capacity as recently shown for new-born wild boars^24^. Nevertheless, the proportions of light juveniles and LAJ females generally increased under favourable conditions. Under these conditions LAJ animals always dominated within their age classes, regardless of the assumed differential survival between heavy and light juveniles. This is mainly because under favourable conditions more juveniles and LAJ females reproduce, which increases the proportion of light juveniles. These light juveniles additionally survive at a higher rate compared with unfavourable conditions. To be conservative we assumed the same difference in winter survival between light and heavy juveniles for both conditions. Data from other species, however, suggest that there might even be an interactive effect between body mass and environmental condition on juvenile survival e.g.^45,47^. Consequently, the difference in survival between heavy and light juveniles might be stronger under unfavourable conditions with low food availability and temperatures compared with favourable conditions. Such an interaction, however, would only enhance our finding of a proportional increase of LAJ females under improving environmental conditions.

Wild boars with low juvenile body mass also remained lighter later in life^20^. Therefore, a proportional increase of LAJ females will result in an overall reduction of average body mass in wild boar populations. Such decreases in body mass in response to a warming climate were observed in various other species^25,48,49^. For the wild boar, however, we here propose for the first time a potential mechanism for such a decrease in average body mass, namely an increased winter survival of light juveniles. Juvenile body mass also has long lasting effects on reproductive success of wild boar females^20^. Consequently, the increased proportion of LAJ females will further cause a reduction in average litter sizes. Hence, our models indicate that, with increasingly favourable environmental conditions, wild boar populations will consist of smaller individuals which give birth to fewer offspring. Those traits are known from Mediterranean wild boar populations^13,40^. Accordingly, by including variation in juvenile body mass, our models revealed a lower asymptotic population growth (λ) under favourable environmental conditions compared with a previous study, while λ was similar under unfavourable conditions (λ = 1.44 and λ = 0.83 versus λ = 1.63 and λ = 0.85 found by Bieber and Ruf^16^).

The increasing importance of LAJ females under favourable environmental conditions is also reflected by their elasticities, i.e., their relative contribution to λ. As in previous models^16^ we found a crossover in the ranking of elasticities from adult survival under poor to juvenile survival under good conditions. However, here we show that the predominant effect of adult survival under poor conditions almost solely originates from HAJ females. A similar high importance of adults under unfavourable conditions was also found in other large ungulates^14,16,50^.

In favourable environments, LAJ females become almost as important for population growth as HAJ females despite their lower recruitment rates. Although such a high importance of juveniles is very unusual for similar sized ungulates^50,51^ it is in line with previous wild boar population models. This is because of the exceptional life history strategy of wild boars compared with other ungulates, namely their large litter size and their early age at sexual maturity^14,16^. While life history and resulting population dynamics of wild boar are rather peculiar for large ungulates, the methods outlined here will be applicable to other species as well. Especially the hybrid Leslie matrix population model combining an age-class model with a multi-level phenotype variable may be useful for models of any species where differential phenotypic traits affects reproduction and/or survival.

### Effects of culling regimes

Our results indicate a slightly decreased population growth under favourable conditions compared with a previous model that did not include effects of juvenile body mass^16^. However, our current findings of a changing population structure reveal further challenges for wild boar management. There is an ongoing debate on which classes to harvest predominantly in order to achieve an optimal wildlife management in various species^52^ (and references therein). Some authors argue that wildlife managers should mimic natural predation and harvest younger individuals in order to achieve a most natural population structure^9,34,53^. Others reason that this could be best achieved by a totally unselective hunting except for some special scenarios^52^. Some of these scenarios, however, actually apply to the wild boar. For instance, despite the return of the wolf (*Canis lupus*) to some parts of Central Europe^54,55^, many wild boar populations still do not face natural predation. Further, selective hunting could counteract anthropogenic effects like selection for earlier reproduction^52^, which was hypothesised to result from high hunting pressure in wild boars^36^. Finally, selective harvest might also be used to counteract the climate-driven changes in population structure and the resulting phenotypic change identified here.

Our hunting models mimicking natural predation revealed stable stage distributions that were closest to those resulting from an entirely unselective harvest. In contrast to an entirely unselective culling, the natural predation scenarios were additionally characterised by a slightly increased proportion of adult sows within the resulting population. However, strategies focusing more on juveniles inevitably were also characterised by higher numbers of animals that needed to be harvested. This effect is even enhanced by the poor discriminability between male and female juveniles from a distance, i.e. during normal hunting circumstances. Given increasing human-wildlife conflicts and ecological threats caused by exponentially growing wild boar populations, a limitation of population growth or even a population reduction is urgently required in many areas^9,13,27–29^. Therefore, an important objective of a selective harvest in wild boar could be to maximise hunting efficiency in order to bring down population growth^52^.

Our models revealed that, under an entirely unselective culling regime, about 30% of all females need to be harvested per year to achieve a stable population size. To identify more efficient strategies, actual numbers of animals rather than model-elasticities alone have to be considered. Juveniles proved to be very important with respect to population growth under good conditions. However, due to the high proportion of this age class within the population, focusing on juveniles would require considerably more individuals to be culled. This is also true for hunting models mimicking natural predation with low proportions of adults and yearlings harvested^43^, although their efficiency could be improved by targeting predominantly heavy juveniles.

In contrast, the highest efficiency in terms of the required number of harvested individuals is reached by targeting mainly yearlings and adults. Removing a single individual of these classes affects population growth 3.6 times more strongly than harvesting a light juvenile. However, employing a pure strategy, 100% of adult sows would need to be harvested to achieve a barely stable population (λ = 0.97), which seems a rather unrealistic goal. This reinforces the finding by Bieber and Ruf^16^ that strategies focusing on adults alone are not sufficient to effectively reduce wild boar populations under favourable environmental conditions.

Interestingly, our results show that harvesting yearling females is as effective as targeting adult sows. Furthermore, a population reduction can be achieved by harvesting 80% of the yearling females. This corresponds to less than 20% of all females in our idealised population, if targeted alone. This finding is in line with previous pure age-based or body mass-based population models. Those models revealed a high impact of increased culling of medium sized or yearling females on wild boar population growth^14,22^. Unlike in juveniles, sex discrimination from a distance is not a problem in yearlings, which reduces hunting effort enormously. Further, differences caused by previous juvenile body mass might be still more visible in yearlings compared to adult sows. Therefore, the efficiency of such a yearling-based strategy may be further increased by targeting predominantly heavy yearlings.

However, an increased hunting pressure on yearlings caused a population structure with an even lower proportion of adult females than found without hunting. Thus, there is a trade-off between hunting efficiency and the maintenance of a more natural population structure, which would require a higher harvest rate of juveniles. Such a scenario is presented in our natural hunting model. This strategy, however, should only be applied if hunting pressure and success is high enough to guarantee a sufficient reduction of wild boar population growth.

## Supplementary information

Supplementary Information.

## Data Availability

All data used are publically available from the literature and explicitly shown in Table 2 with the respective references.
