# Supplementary material for: How climate change and wildlife management affect population structure in wild boars
Source: Sci Rep. 2020 Apr 29;10:7298. doi: 10.1038/s41598-020-64216-9 (PMC7190818; doi:10.1038/s41598-020-64216-9)
Supplement: Supplementary file 1 — Supplementary Information. [file 41598_2020_64216_MOESM1_ESM.pdf]

How climate change and wildlife management affect population structure in wild boar

Sebastian G. Vetter, Zsófia Puskas, Claudia Bieber, Thomas Ruf

Supplementary Information 1

Detailed information on the data used to parameterise the models for favourable and unfavourable environmental conditions.

Fecundity

Fecundity is the average number of females produced by a female of a certain age, e.g.[1,2]. It was calculated using literature data on mean number of foetuses corrected for intrauterine mortality and breeding probability (BP). Due to the extended reproduction and parturition period in wild boar, the correct proportion of reproducing females is difficult to determine[3]. Most studies only consider the proportion of breeding females during the hunting period, e.g.[4]. Extensively reviewing the existing literature, Briedermann[3] concluded that about 100 % of yearling and adult females are reproductively active throughout the year, which therefore was also assumed in the present model regardless of environmental conditions. Most recent data on juvenile reproduction were reported in Gethöffer, et al.[5] and Servanty, et al.[4]. For juveniles with more than 25 kg of dressed body mass (i.e., heavy juveniles) Gethöffer, et al.[5] report juvenile BPs of 60 % and 43 % under favourable and unfavourable conditions, respectively, in Eastern Lower Saxony. According to Briedermann[3] this study site was most comparable to his study population from which survival rates were taken (see below). Therefore, these values were used in the present model for juveniles although in other areas juvenile BP might be up to 85 %, likely due to regional differences[5]. Data especially on light juveniles can be found in Servanty, et al.[4] who reported a BP of 40 % and 20 % for juveniles between 20 and 25 kg dressed body mass in favourable and unfavourable conditions, respectively. However, the proportion of juveniles below 20 kg remained unmentioned [4]. Including these light juveniles likely reduces the reported percentages considerably as juveniles above 20 kg dressed body mass only represent the most upper part of what was considered to be light juveniles in the present model (threshold of about 30 kg live weight, see below). Because of this, BP of light juveniles here was set to 10 % and 0 % under favourable and unfavourable conditions, respectively. This is in line with data from Lower Saxony where juveniles below 25 kg were not found to be reproductively active at all[5].

Although the average number of foetuses might have generally increased over the past decades[3], no recent detailed data on number of foetuses was available with respect to both age class and environmental conditions. Therefore, the values reported in Briedermann[3] were used in the present model (i.e., 4.6, 6.7, 6.8 and 3.2, 5.4, 6.2 foetuses for juveniles, yearlings, and adults under favourable and unfavourable conditions, respectively). Nevertheless, these values are comparable to more recent data reported from other populations[5,6]. The numbers of foetuses were corrected using the only complete age-class specific data published on

intrauterine mortalities; i.e., 11 %, 18 %, and 6 % for juveniles, yearlings and adults, respectively[5].

Differences in the number of offspring depending on maternal juvenile body mass were not available from the literature. The same was true for the relative contribution to light (LJ) and heavy juveniles (HJ) by LAJ and HAJ females, respectively. These proportions were therefore calculated from our own data on reproductive success of semi-naturally kept yearling and adult females collected in 2013 and 2015, respectively (for details on the two study populations and their housing condition see[7]). Due to limited supplementary feeding representing intermediate food availability in 2014[7], data from this year were not considered in the present analysis. Food availability in 2013 and 2015 corresponded to favourable environmental conditions in the present study (for details see[7]). To calculate the mean number of offspring produced LAJ and HAJ females as well as their respective proportional contribution to LJ and HJ, F0-females of the cohort 2011 were classified according their median juvenile body mass at around 8 months of age (31.2 kg).

In 2013, when F0-females reproduced as yearlings, females classified as yearlings light as juveniles (Y_{LAJ}, n = 20) produced 15.7 % less offspring than the overall mean in 2013 (2.14). Females identified as yearlings heavy as juveniles (Y_{HAJ}, n = 21) produced 16.5 % more offspring compared to the overall average. In 2015, when F0-females reproduced as adults, females classified as adults light as juveniles (A_{LAJ}, n = 23) produced 6.5 % less offspring than the overall mean in 2015 (2.65). Females heavy as juveniles (A_{HAJ}, n = 20) produced 9.0 % more compared to the average. For juveniles the same percentages were assumed as those found in yearlings due to a lack of data[7].

The proportional production of light or heavy juveniles by LAJ and HAJ females was calculated from the same data[7]. Offspring of F0-females (F1 born in 2013 and 2015) were classified as light and heavy juveniles based on the median of juvenile body mass of piglets born in 2015 (21.5 kg; i.e., piglets born by adult sows). This threshold was used to classify F1-juveniles from both years to exclude the effect of mother´s young age in 2013. This was necessary as piglets born by yearlings were on average lighter compared with those born by adult sows (mean ± sd = 18.8 ± 5.1 kg (n = 112) and 21.7 ± 5.3 kg (n = 112) in 2013 and 2015, respectively; t = 4.19, $P < 0.001$). A different threshold was used compared with the median body mass from F0-females because F1-piglets in 2013 and 2015 were weighed as young as six months and thus about two months earlier than the F0-females in 2011. In 2013, when F0-females were yearlings, 35.7 % and 26.2 % of offspring of F0-females light (Y_{LAJ}) or heavy (Y_{HAJ}) as juveniles were classified as heavy juveniles, respectively (Supporting Table S2). In 2015, when F0-females were adults 28.0 % and 73.9 % of the offspring of adult F0-females light (A_{LAJ}) or heavy (A_{HAJ}) as juveniles were classified as heavy juveniles, respectively. Given that females reproduce earlier in mast years and that body mass gain during the first year of life is linear[3], it is plausible to assume that females contribute in higher proportions to heavy juveniles under favourable conditions. Therefore, a 20 % divergence from the computed values in favour to the production of light juveniles was assumed under unfavourable conditions.

Survival

Annual survival rates were taken from Briedermann[3] as those were based on a large database collected over several decades and provide survival data for different environmental scenarios. Seasonal survival rates of yearling and adult female wild boars were not available from the literature. The main natural cause of death among older wild boar classes is starvation[8], which occurs mostly in winter when food availability is lowest. Consequently, a high summer survival of 95 % was assumed for yearlings and adults. Validation of this assumption revealed no major effect on the model outcome (see Results and Supplementary Information 2). For juveniles some studies report postnatal or summer survival rates. Excluding the artificial enclosure situation reported in Martys[9] and the exceptionally high mortality rate found in an Hungarian population[3,10], we used the juvenile summer survival rate of 73 % as reported by Fruzinski[11] for Polish juveniles. The Polish population studied by Fruzinski[11] in fact is geographically close and thus very similar to the East German population reported in Briedermann[3].

Further, no literature data were available regarding the differences in survival in relation to juvenile body mass. Due to a lower body surface to volume ratio and the resulting higher energy demands for thermoregulation, LJ are more susceptible to cold temperatures[12,13]. Although it has never been shown explicitly for wild boars that body mass affects survival of juveniles, it nevertheless seems plausible that LJ have lower survival rates than HJ. Thus, winter survival was assumed to be 10 % higher and lower for HJ and LJ, respectively, compared to the averages under favourable and unfavourable conditions. The effect of this assumption was also tested by altering this percentage (Supplementary Information 2). Again, the models were found to be very robust towards changes in this percentage, except for the unlikely scenario that heavy and light juveniles would not differ in their survival rates under unfavourable environmental conditions (Supplementary Information 2). Again, only under unfavourable conditions was the proportion of LAJ and HAJ females also affected by altering the difference in survival between HJ and LJ (Supplementary Information 2). Neither of these, however, affected the conclusions we drew from the model outcome (see also main text).

With increasing body size, relative difference in the surface to volume ratio, and thus the differences in energy demands for thermoregulation[12], diminish. Therefore, no difference in survival of yearlings and adults based on their juvenile body mass was assumed. Although potential long-term effects of juvenile body mass on survival, as found for reproduction[7], cannot be excluded finally, it seems plausible that the reduced reproductive effort in females which have been light as juveniles (see above) results from a trade-off between survival and reproduction[14] and thus causes no long-term effect of juvenile body mass on survival.

Because winter conditions are more strongly affected by climate change in temperate regions[15,16] compared to summer conditions, the two different parameterisations of the model (i.e., favourable and unfavourable) refer only to winter conditions. Therefore, identical summer survival rates of all classes were used in both models. This is in line with a very weak and so far negligible negative effect of increasing summer temperatures on wild boar population growth[17]. Winter survival rates were calculated from annual and summer survival rates ($S_{winter} = S_{annual} / S_{summer}$).

Supplementary Table S1 Published survival rates for Central European wild boar populations.

Vital rate	Conditions	Age classes			References
		Piglets	Yearlings	Adults	
Survival (%)		70	70	70	[18]
		73			[11]
		81.8	87.6		[19]
		64-87			[20]
	poor	25	31	58	[2] (data from Briedermann[3], weighted by No. of animals in cohorts)
	intermediate	33	40	66	
	good	52	60	71	
Neonatal survival (%)	favourable	45			[10]
	unfavourable	39			
Summer survival (%)	favourable	94			
	unfavourable	91			

Supplementary Information 2

Validating the effect of model assumptions that had to be drawn due to a lack of empirical data

Two major assumptions had to be drawn during the model construction due to the lack of empirical data: (i) on the summer survival of yearlings and adults, which was set to 95 % (see main article) and (ii) on the difference in annual survival rates of light (LJ) and heavy juveniles (HJ), which was set to 20 %. In order to test for the effect of these assumptions on the results, and thus on the conclusions from the population modelling, these percentages were varied and the different model outcomes were compared with respect to sensitivities, elasticities, and stable stage distributions.

Varying summer survival of yearlings and adults

For validation of the assumed summer survival rates of yearling and adults this rate was modified from 85 % to 100 % (model assumption: 95 %).

Sensitivities

Varying summer survival among yearlings and adults had virtually no effect on sensitivities, neither under favourable nor under unfavourable conditions. With decreasing summer survival of adults and yearlings, the sensitivity of juvenile survival increased only slightly (Fig. S1 and Fig. S2) and no changes regarding the ranking of sensitivities were caused.

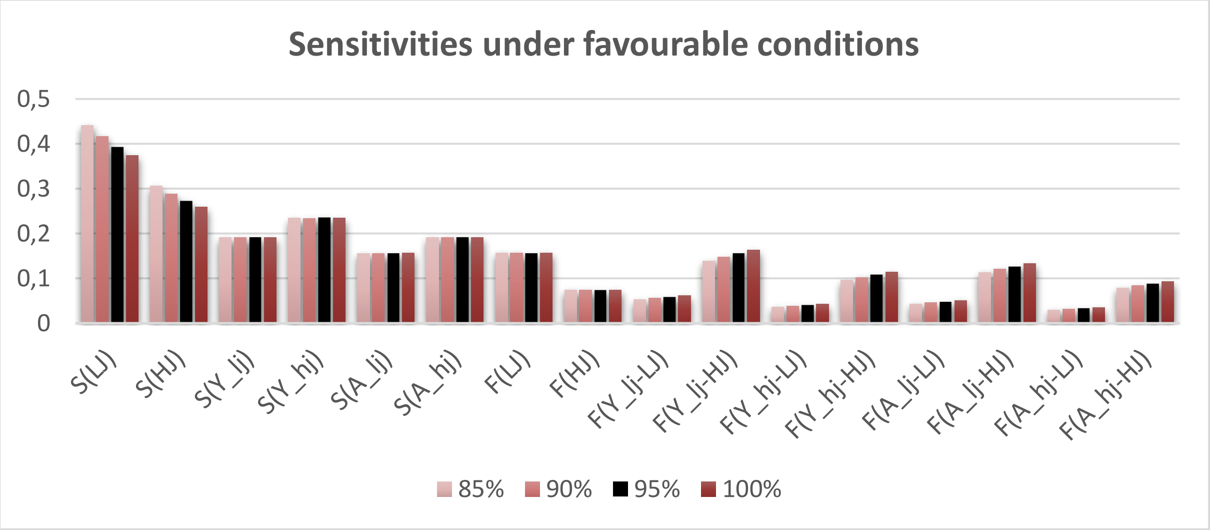

Figure S1 Sensitivities under favourable conditions with varying summer survival rates of yearlings and adults. Vital rates are survival (S) and fecundity (F) of the age classes in brackets: heavy juveniles (HJ), light juveniles (LJ), yearlings heavy (Y_hj) and light as juveniles (Y_lj), and adults heavy (A_hj) and light as juveniles (A_lj). Yearlings and adults can contribute to both heavy and light juveniles; the contribution is signed by a hyphen (e.g., Y_lj-HJ).

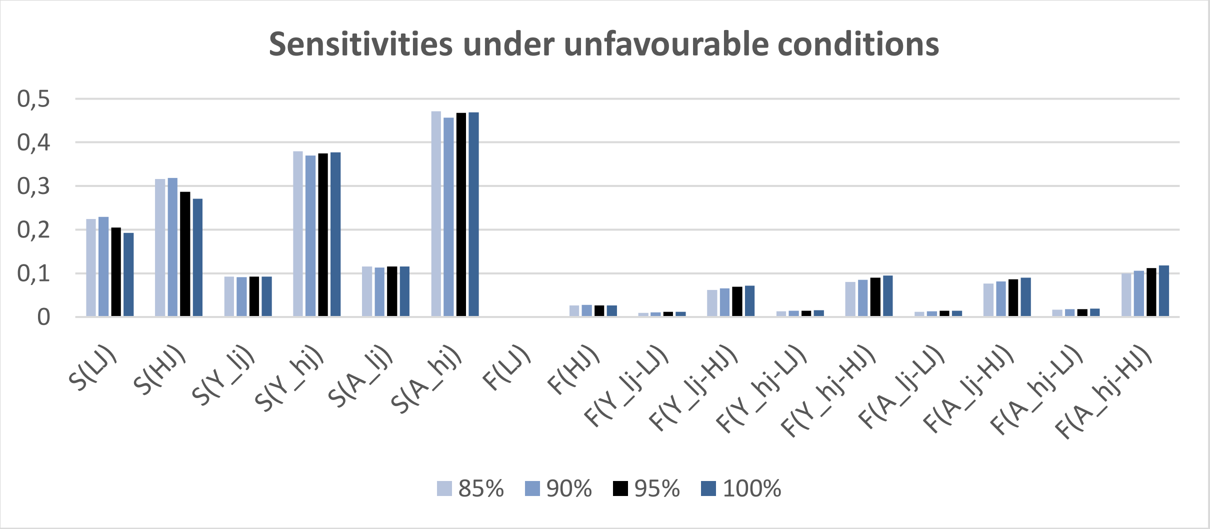

Figure S2 Sensitivities under unfavourable conditions with varying summer survival rates of yearlings and adults. Vital rates are survival (S) and fecundity (F) of the age classes in brackets: heavy juveniles (HJ), light juveniles (LJ), yearlings heavy (Y_hj) and light as juveniles (Y_lj), and adults heavy (A_hj) and light as juveniles (A_lj). Yearlings and adults can contribute to both heavy and light juveniles; the contribution is signed by a hyphen (e.g., Y_lj-HJ).

Elasticities

Elasticities were even less affected than sensitivities by varying summer survival among yearlings and adults, regardless of environmental conditions (Fig. S3 and Fig. S4).

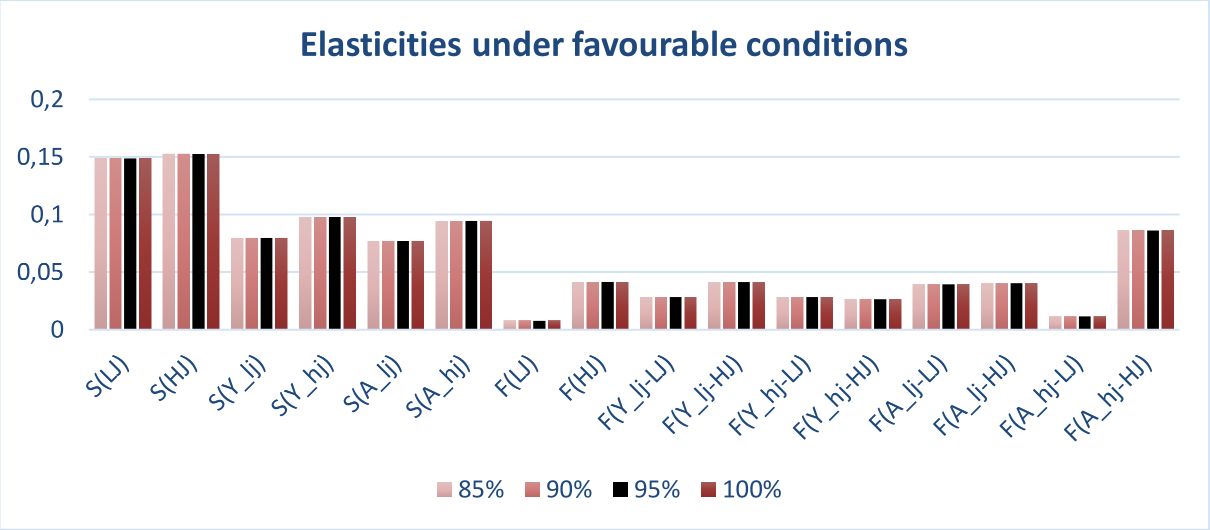

Figure S3 Elasticities under favourable conditions with varying summer survival rates of yearlings and adults. Vital rates are survival (S) and fecundity (F) of the age classes in brackets: heavy juveniles (HJ), light juveniles (LJ), yearlings heavy (Y_hj) and light as juveniles (Y_lj), and adults heavy (A_hj) and light as juveniles (A_lj). Yearlings and adults can contribute to both heavy and light juveniles; the contribution is signed by a hyphen (e.g., Y_lj -HJ).

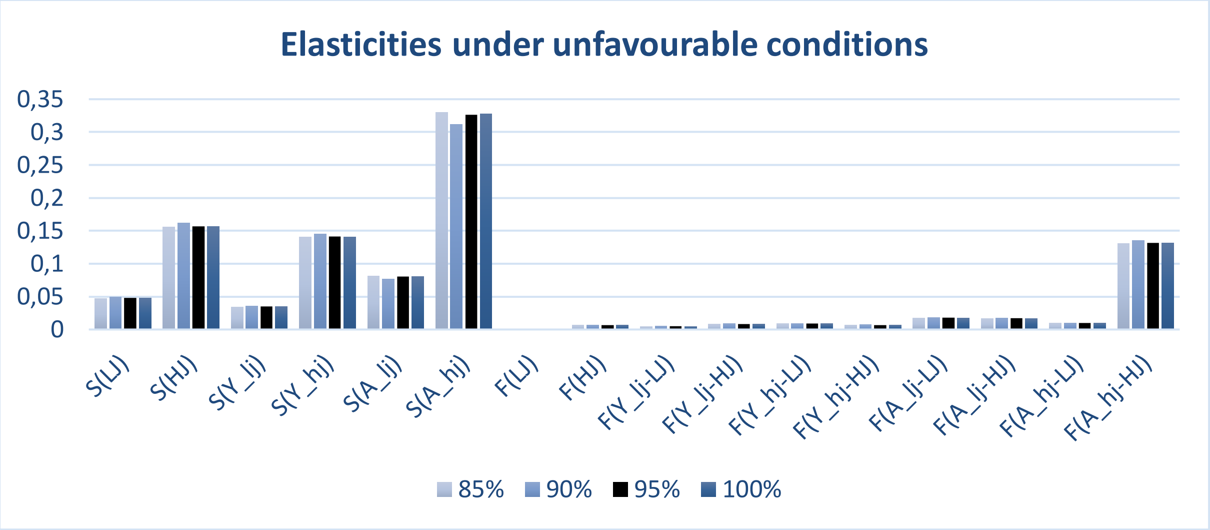

Figure S4 Elasticities under favourable conditions with varying summer survival rates of yearlings and adults from. Vital rates are survival (S) and fecundity (F) of the age classes in brackets: heavy juveniles (HJ), light juveniles (LJ), yearlings heavy (Y_hj) and light as juveniles (Y_lj), and adults heavy (A_hj) and light as juveniles (A_lj). Yearlings and adults can contribute to both heavy and light juveniles; the contribution is signed by a hyphen (e.g., Y_lj-HJ)

Population structure

Finally, there was also only a minor effect on population structure of varying the rate of summer survival of yearlings and adults. Decreasing their summer survival caused only slight increases in the proportion of juveniles (both classes) whereas the proportion of yearlings and adults slightly decreased under both conditions (Fig. S5 and Fig. S6).

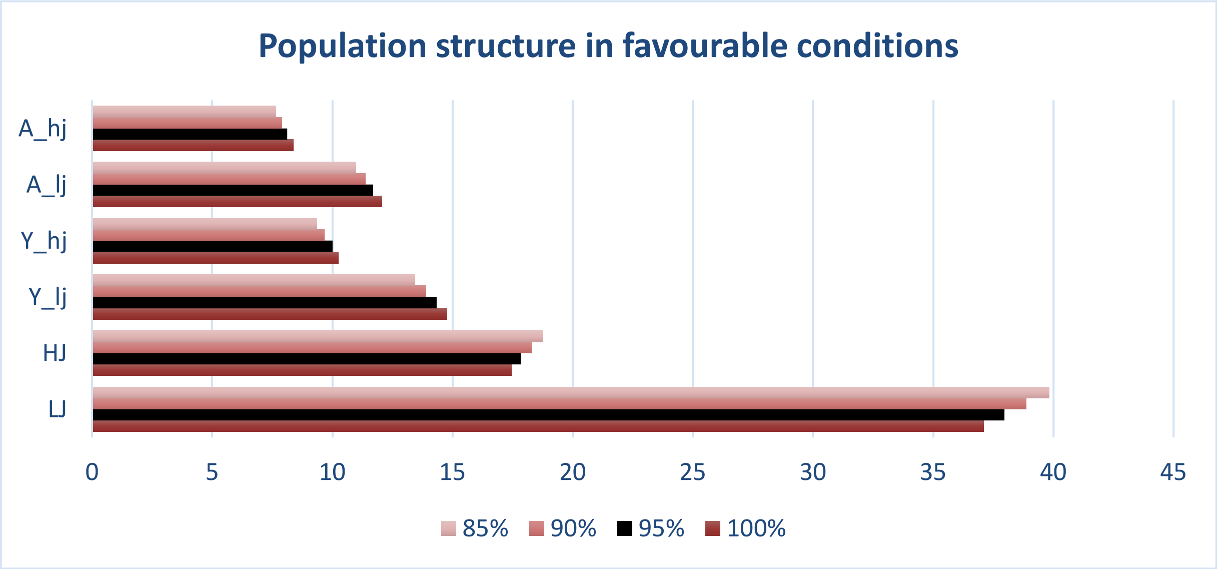

Figure S5 Stable stage distribution under favourable conditions with varying summer survival rates of yearlings and adults. Age classes are heavy juveniles (HJ), light juveniles (LJ), yearlings heavy (Y_hj) and light as juveniles (Y_lj), and adults heavy (A_hj) and light as juveniles (A_lj).

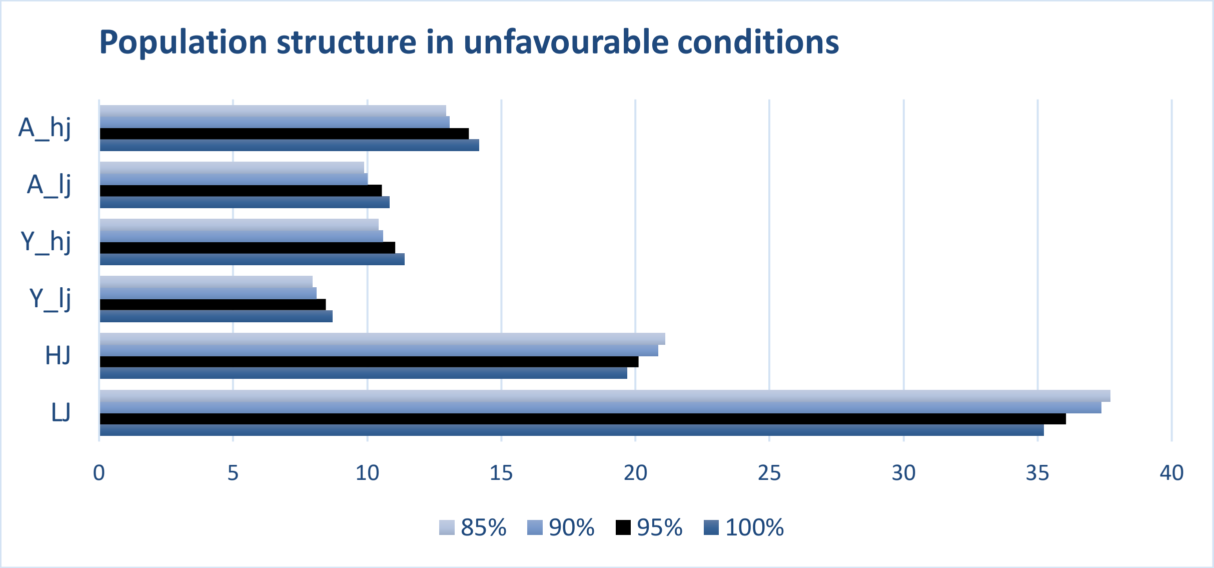

Figure S6 Stable stage distribution under unfavourable conditions with varying summer survival rates of yearlings and adults. Age classes are heavy juveniles (HJ), light juveniles (LJ), yearlings heavy (Y_hj) and light as juveniles (Y_lj), and adults heavy (A_hj) and light as juveniles (A_lj).

Varying differences in winter survival rates of juveniles

For validation, the assumed difference in winter survival rates between HJ and LJ was varied from ± 0 % to ± 15 % in steps of 5 % (model assumption: ± 10 % from the overall mean for HJ and LJ, respectively; see main article).

Sensitivities

Varying differences in survival rates between LJ and HJ did not notably affect the sensitivities under favourable conditions and thus the conclusions drawn from the models. The higher the differences in survival rates between HJ and LJ the more importance heavy juveniles gained under unfavourable conditions. This, however, did not affect the conclusions as the survival of light juveniles always had the highest sensitivity, irrespective of the difference in survival between the two juvenile classes (Fig. S7). Also, under unfavourable conditions, varying the difference in annual survival between HJ and LJ only caused minor differences on the sensitivities. Except for the assumption that there is no difference (i.e., ± 0 %), in which case survival of LJ showed the highest sensitivity, survival of adults heavy as juveniles (A_hj) always showed the highest sensitivity (Fig. S8). Arguably, under unfavourable environmental conditions with cold winters and low food availability a lower body mass is especially detrimental[21]. Therefore, the assumption that HJ and LJ do not differ in their survival seems highly unlikely so that also under unfavourable conditions the main conclusions drawn from the model results remain largely unaffected.

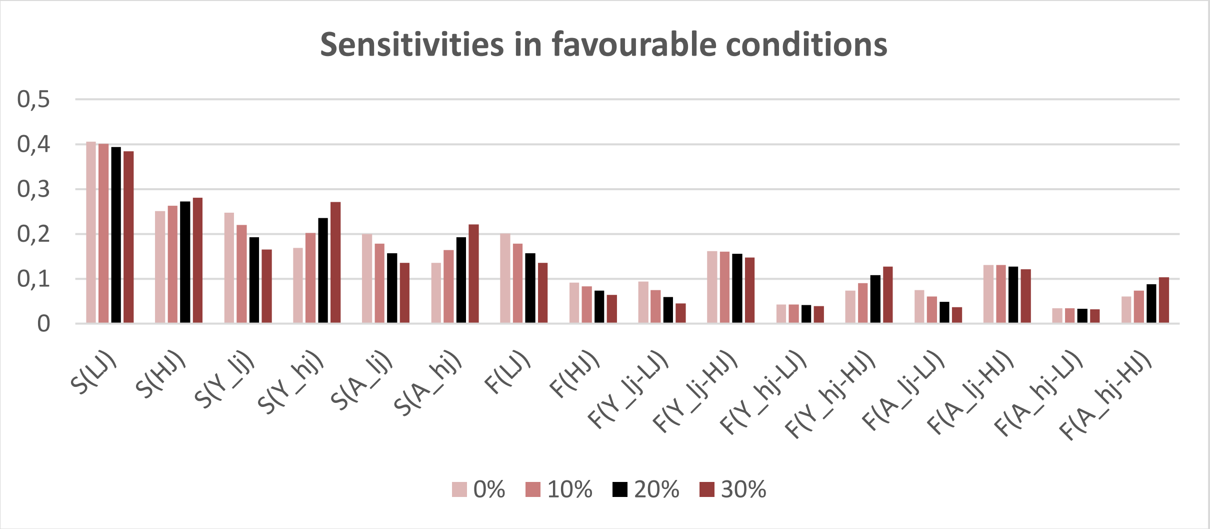

Figure S7 Sensitivities under favourable conditions with varying differences in yearly survival rates between heavy and light juveniles. Vital rates are survival (S) and fecundity (F) of the age classes in brackets: heavy juveniles (HJ), light juveniles (LJ), yearlings heavy (Y_hj) and light as juveniles (Y_lj), and adults heavy (A_hj) and light as juveniles (A_lj). Yearlings and adults can contribute to both heavy and light juveniles; the contribution is signed by a hyphen (e.g., Y_lj-HJ)

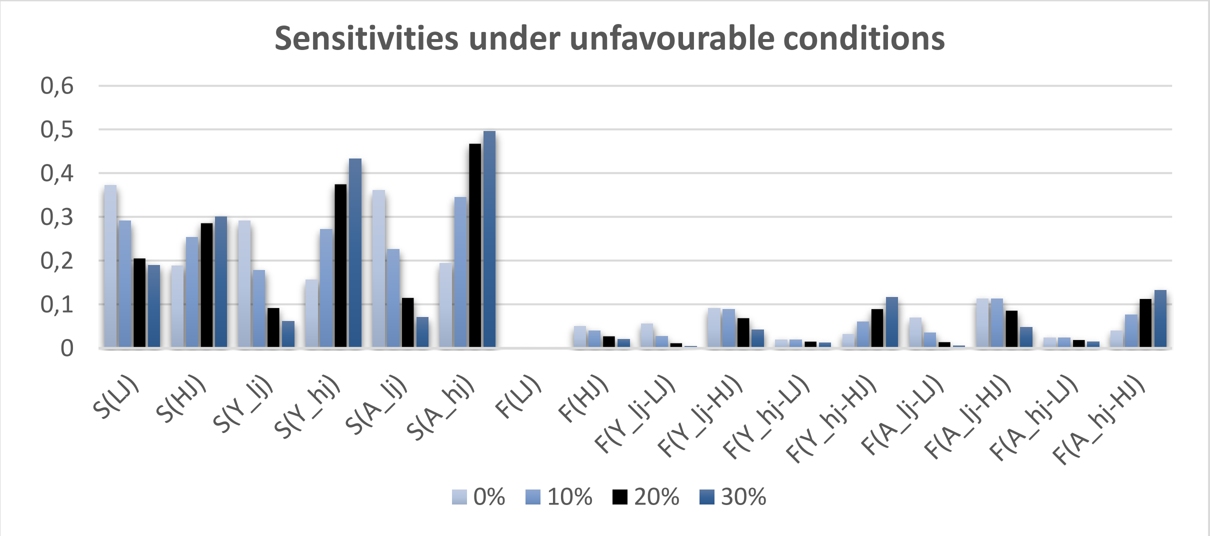

Figure S8 Sensitivities under unfavourable conditions with varying differences in yearly survival rates between heavy and light juveniles. Vital rates are survival (S) and fecundity (F) of the age classes in brackets: heavy juveniles (HJ), light juveniles (LJ), yearlings heavy (Y_hj) and light as juveniles (Y_lj), and adults heavy (A_hj) and light as juveniles (A_lj). Yearlings and adults can contribute to both heavy and light juveniles; the contribution is signed by a hyphen (e.g., Y_lj-HJ).

Elasticities

Similar to sensitivities, increasing the difference in annual survival between HJ and LJ increased the importance of individuals heavy as juveniles with respect to elasticities. Again, these changes did not majorly affect the conclusions drawn from the models as the class with the highest elasticity did not change under both favourable (i.e., survival of both juvenile classes) and unfavourable (i.e., survival of A_hj) conditions. As with sensitivities, the only exception was the unlikely assumption of no difference in annual survival between HJ and LJ under unfavourable environmental conditions (Fig. S9 and Fig. S10).

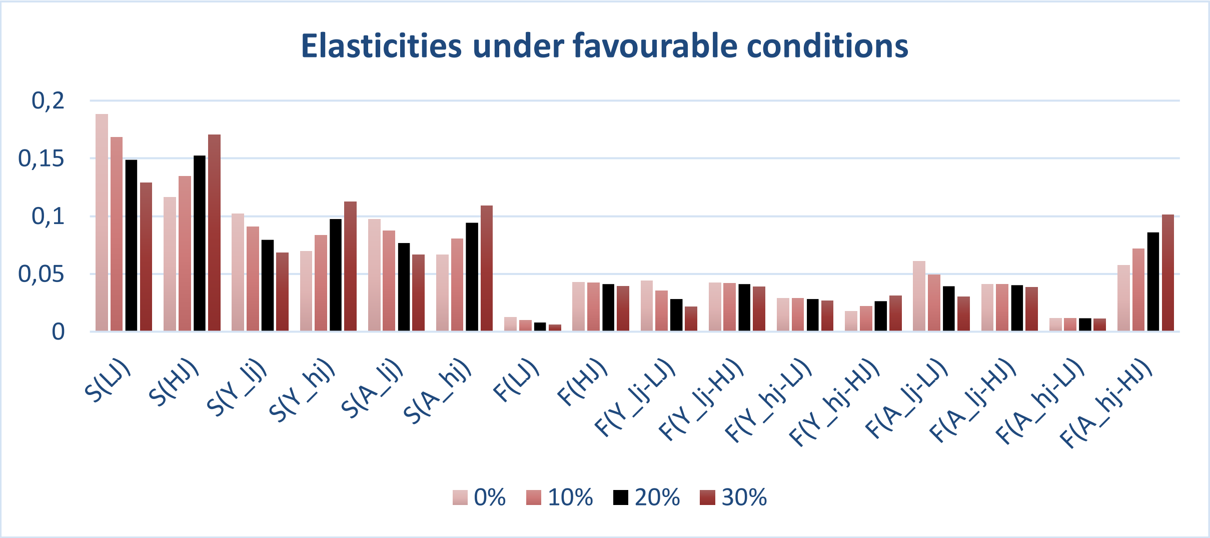

Figure S9 Elasticities under favourable conditions of versions, varying the differences in yearly survival rates between heavy and light juveniles from 0 % to 15 %. Vital rates are survival (S) and fecundity (F) of the age classes in brackets: heavy juveniles (HJ), light juveniles (LJ), yearlings heavy (Y_hj) and light as juveniles (Y_lj), and adults heavy (A_hj) and light as juveniles (A_lj). Yearlings and adults can contribute to both heavy and light juveniles; the contribution is signed by a hyphen (e.g., Y_lj-HJ).

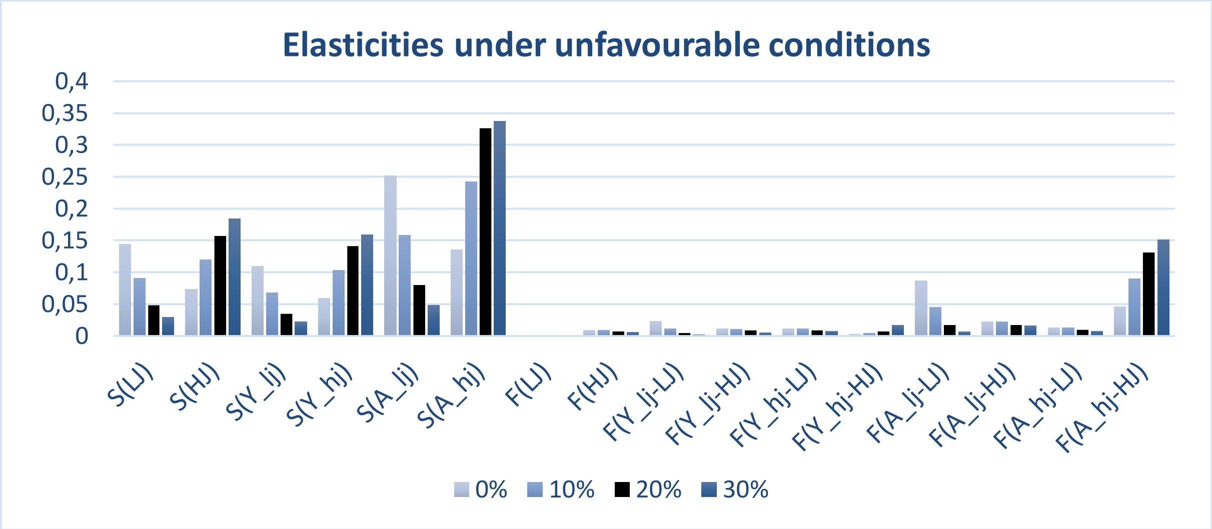

Figure S10 Elasticities under unfavourable conditions with varying the differences in yearly survival rates between heavy and light juveniles. Vital rates are survival (S) and fecundity (F) of the age classes in brackets: heavy juveniles (HJ), light juveniles (LJ), yearlings heavy (Y_hj) and light as juveniles (Y_lj), and adults heavy (A_hj) and light as juveniles (A_lj). Yearlings and adults can contribute to both heavy and light juveniles; the contribution is signed by a hyphen (e.g., Y_lj-HJ).

Population structure

The age structure of the asymptotic population, as well as the stable stage structure within the age classes, also remained largely unaffected by varying the difference in annual survival of HJ and LJ. The proportion of individuals light as juveniles increased slightly with decreasing survival differences (Fig. S11) but no qualitative changes could be observed and all age classes were dominated by individuals light as juveniles under favourable conditions. Under unfavourable conditions, however, this effect was stronger and caused qualitative changes. If the assumed difference in survival was below 20 % all age classes were again dominated by individuals light as juveniles. Otherwise older classes (i.e., yearlings and adults) were dominated by individuals heavy as juveniles (Fig. S12).

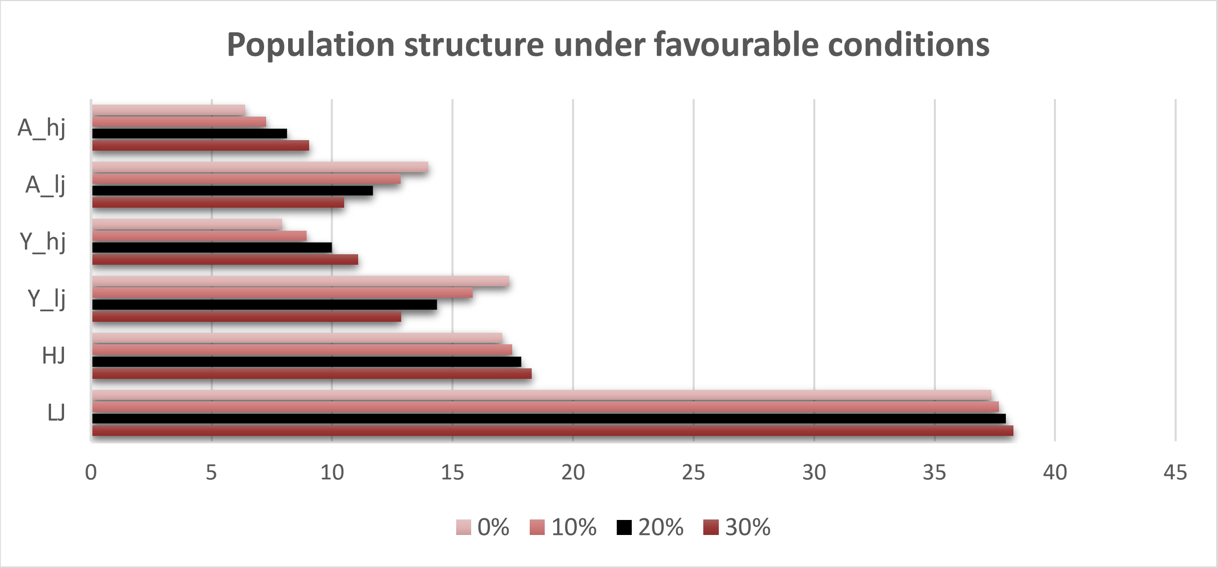

Figure S11 Stable stage distribution under favourable conditions with varying differences in yearly survival rates between heavy and light juveniles. Heavy juveniles (HJ), light juveniles (LJ), yearlings heavy (Y_hj) and light as juveniles (Y_lj), and adults heavy (A_hj) and light as juveniles (A_lj).

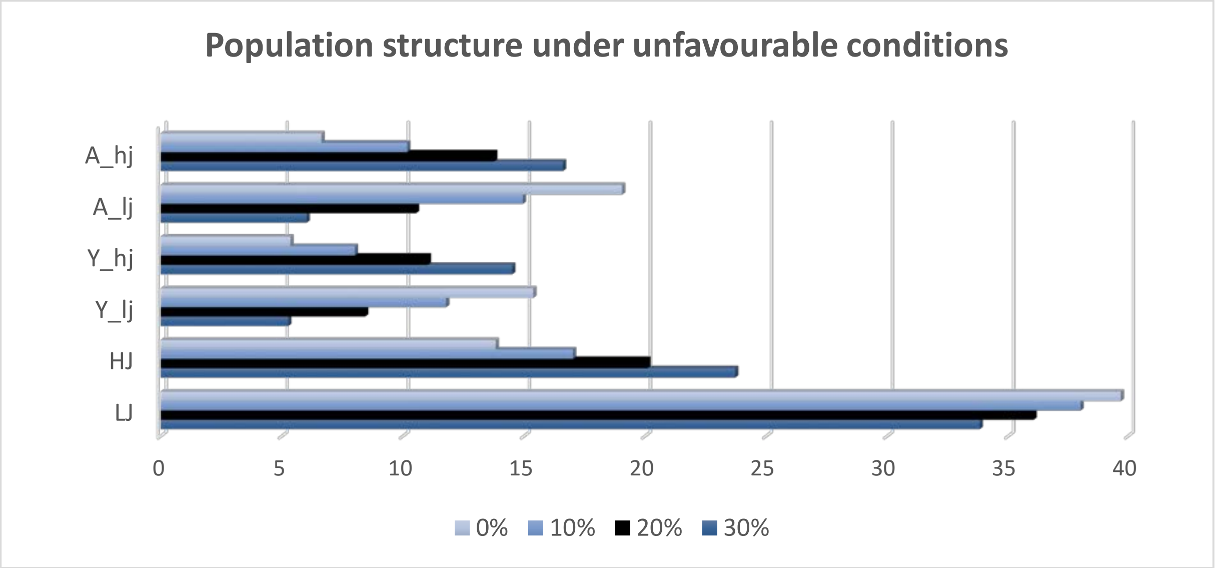

Figure S12 Stable stage distribution under unfavourable conditions with varying differences in yearly survival rates between heavy and light juveniles. Heavy juveniles (HJ), light juveniles (LJ), yearlings heavy (Y_hj) and light as juveniles (Y_lj), and adults heavy (A_hj) and light as juveniles (A_lj).

Supplementary Table S3

Supplementary Table S2 Relative effect of the removal of a single individual of a given class on population growth in relation to that of the removal of a light juvenile (i.e., the class with the lowest effect).

Class	Sensitivity of survival	Proportion in population	Weighted sensitivity	Corrected for discriminability	effect of a single removal
LJ	0.3931	0.380	1.034	0.517	1.0
HJ	0.2726	0.179	1.523	0.761	1.5
Y_{LAJ}	0.1921	0.144	1.334	1.846	3.6
Y_{HAJ}	0.2357	0.100	2.357	1.846	3.6
A_{LAJ}	0.1565	0.117	1.338	1.855	3.6
A_{HAJ}	0.1921	0.081	2.372	1.855	3.6

The calculation was based on the survival sensitivities of the respective age classes weighed by the proportion of the classes within the stable age population, respectively. The classes are light (LJ) and heavy juveniles (HJ), yearlings light (Y_{LAJ}) and heavy as juveniles (Y_{HAJ}) as well as adults light (A_{LAJ}) and heavy as juveniles (A_{HAJ}). Weighted sensitivities were additionally corrected for discriminability as follows: For LJ and HJ weighed sensitivities were divided by 2 as distinguishing between male and female piglets is difficult and one needs to assume that by chance only every second animal shot is a female; for yearlings and adults weighed sensitivities were averaged across individuals that have been light or heavy at juveniles as those are might not easily distinguishable anymore in these age classes.

Supplementary References

1 Servanty, S. *et al.* Influence of harvesting pressure on demographic tactics: implications for wildlife management. *J Appl. Ecol.* **48**, 835-843, doi:10.1111/j.1365-2664.2011.02017.x (2011).

2 Bieber, C. & Ruf, T. Population dynamics in wild boar *Sus scrofa*: ecology, elasticity of growth rate and implications for the management of pulsed resource consumers. *J Appl. Ecol.* **42**, 1203-1213, doi:10.1111/j.1365-2664.2005.01094.x (2005).

3 Briedermann, L. *Schwarzwild*. 2nd edn, (Franckh-Kosmos Verlags-GmbH & Co. KG, 2009).

4 Servanty, S., Gaillard, J.-M., Toïgo, C., Brandt, S. & Baubet, E. Pulsed resources and climate-induced variation in the reproductive traits of wild boar under high hunting pressure. *J Anim. Ecol.* **78**, 1278-1290, doi:10.1111/j.1365-2656.2009.01579.x (2009).

5 Gethöffer, F., Sodeikat, G. & Pohlmeyer, K. Reproductive parameters of wild boar (*Sus scrofa*) in three different parts of Germany. *Eur. J Wildl. Res.* **53**, 287-297, doi:10.1007/s10344-007-0097-z (2007).

6 Servanty, S., Gaillard, J.-M., Allainé, D., Brandt, S. & Baubet, E. Litter size and fetal sex ratio adjustment in a highly polytocous species: the wild boar. *Behav. Ecol.* **18**, 427-432, doi:10.1093/beheco/arl099 (2007).

7 Vetter, S. G. *et al.* Shy is sometimes better: personality and juvenile body mass affect adult reproductive success in wild boars, *Sus scrofa*. *Anim. Behav.* **115**, 193-205, doi:10.1016/j.anbehav.2016.03.026 (2016).

8 Okarma, H., Jędrzejewska, B., Jędrzejewski, W., Krasinski, Z. A. & Milkowski, L. The roles of predation, snow cover, acorn crop, and man-related factors on ungulate mortality in Białowieża Primeval Forest, Poland. *Acta Theriol.* **40**, 197-217 (1995).

9 Martys, M. Gehegebeobachtungen zur Geburts- und Reproduktionsbiologie des Europäischen Wildschweines (*Sus scrofa* L.). *Z Säugetierk.* **47**, 100-113 (1982).

10 Náhlik, A. & Sándor, G. Birth rate and offspring survival in a free-ranging wild boar *Sus scrofa* population. *Wildl. Biol.* **9**, 249-254, doi:10.2981/wlb.2003.062 (2003).

11 Fruzinski, B. Situation of wild boar populations in western Poland. *IBEX J.M.E.* **3**, 186-187 (1995).

12 Kleiber, M. Body size and metabolic rate. *Physiol. Rev.* **27**, 511–541, doi:10.1152/physrev.1947.27.4.511 (1947).

13 Nowack, J. *et al.* Muscle nonshivering thermogenesis in a feral mammal. *Sci. Rep.* **9**, 6378, doi:10.1101/377051 (2019).

14 Fabian, D. & Flatt, T. Life History Evolution. *Nature Education Knowledge* **3**, 24 (2012).

15 Alcamo, J. *et al.* in *Climate Change 2007: Impacts, Adaptation and Vulnerability. Contribution of Working Group II to the Fourth Assessment Report of the Intergovernmental Panel on Climate Change* (eds M.L. Parry *et al.*) Ch. 12, 541-580 (Cambridge University Press, 2007).

16 Walther, G.-R. *et al.* Ecological responses to recent climate change. *Nature* **416**, 389-395, doi:10.1038/416389a (2002).

17 Vetter, S. G., Ruf, T., Bieber, C. & Arnold, W. What Is a Mild Winter? Regional Differences in Within-Species Responses to Climate Change. *PLoS ONE* **10**, e0132178, doi:10.1371/journal.pone.0132178 (2015).

18 Focardi, S., Gaillard, J.-M., Ronchi, F. & Rossi, S. Survival of wild boars in a variable environment: unexpected life-history variation in an unusual ungulate. *J Mammal.* **89**, 1113-1123, doi:10.1644/07-mamm-a-164.1 (2008).

19 Toïgo, C., Servanty, S., Gaillard, J.-M., Brandt, S. & Baubet, E. Disentangling Natural From Hunting Mortality in an Intensively Hunted Wild Boar Population. *J Wildl. Manage.* **72**, 1532-1539, doi:10.2193/2007-378 (2008).
20 Servanty, S. *et al.* Assessing whether mortality is additive using marked animals: a Bayesian state-space modeling approach. *Ecology* **91**, 1916-1923, doi:10.1890/09-1931.1 (2010).
21 Bergmann, C. *Ueber die Verhältnisse der Wärmeökonomie der Thiere zu ihrer Grösse*. (Abgedruckt aus Göttinger Studien 1847. Vandenhoeck und Ruprecht, 1848).
